# At Omaha

**Published:** 1898-09

**Authors:** 


### AT OMAHA.

### THIRTY-FIFTH ANNUAL MEETING OF THE U. S.V. M. A., SEPTEMBER 6, 7, 8.

*Members Present:* Drs. C. A. Cary, Auburn, Ala.; J. C. Norton, Phœnix, Ari.; A. J. Savage, Colorado Springs, Col.; C. B. Robinson, D. E. Salmon, Washington, D. C.; A. H. Baker, L. A. Merillat, Chicago, Ill.; J. R. Mitchell, Evansville, Ind.; T. A. Bown, Chariton; F. H. P. Edwards, Iowa City; G. A. Johnson, Sioux City; John McBirney, Clarinda; J. Miller, Ottumwa; G. A. Scott, Independence; L. U. Shipley, Sheldon; M. Stalker, Ames; H. E. Talbot, Des Moines; G. M. Walrod, Storm Lake; S. S. Whitbeck, Decorah, Iowa; S. L. Hunter, Ft. Leavenworth; C. B. McClelland, Lawrence; S. Stewart, Kansas City, Kansas; J. W. Jameson, Paris, Kentucky; C. W. Heitzman, New Orleans, La.; W. J. Hinman, Winnipeg, Manitoba; A. W. Clement, Baltimore, Md.; S. Brenton, Detroit; Geo. W. Dunphy, Quincy, Michigan; J. N. Gould, Fairmont; C. C. Lyford, Minneapolis; M. H. Reynolds, St. Anthony's Park, Minnesota; John W. Connoway, Columbia; John Forbes, St. Joseph; T. E. White, Columbia, Mo.; M. E. Knowles, Helena, Montana; C. M. Day, A. T. Peters, Lincoln; Don C. Ayer, H. L. Ramacciotti, Omaha, Neb.; Wm. Herbert Lowe, Paterson, N. J.; R. R. Bell, New York City; W. H. Kelly, Albany; James Law, W. L. Williams, Ithaca, New York; W. Horace Hoskins, Leonard Pearson, Philadelphia, Pa.; T. Bent Cotton, Mt. Vernon, Ohio; T. A. Bray, El Paso, Texas; S. B. Nelson, Pullman, Wash.

*New Members Present:* Drs. E. M. Nighbert, Mt. Sterling, Ill.; J. I. Gibson, Denison, Iowa; John S. Anderson, Seward; L. P. Beechey, G. R. Young, Omaha; J. J. Drasky, Crete; Fred. Evans, Grand Island; V. Schaefer, Tekamah; John D. Sprague, David City; W. M. Taylor, York, Neb. Chas. E. Cotton, Minneapolis; S. H. Ward, St. Cloud, Minn. H. A. Christmann, Milwaukee.

*Visitors:* Ladies present—Mrs. L. A. Merillat, Mrs. H. Sorby, Chicago, Ill.; Mrs. J. I. Gibson, Denison; Mrs. C. J. Hinkley, Odebolt; Mrs. G. A. Johnson, Sioux City; Mrs. P. O. Koto, Forest City; Mrs. D. H. Miller, Harlan; Mrs. S. S. Whitbeck, Decorah; Mrs. G. M. Walrod, Storm Lake, Iowa. Mrs. S. Stewart, Miss Belle Stewart, Kansas City; Mrs. T. E. White, Columbia, Mo. Mrs. C. M. Day, Mrs. A. T. Peters, Miss Martha Peters,

Miss Alma Peters, Lincoln; Mrs. F. Draski, Crete, Neb. Mrs. William P. Allen, Mrs. W. Horace Hoskins, Philadelphia, Pa. Mrs. T. A. Bray, El Paso, Texas.

*Members of the Profession Present.*—Drs. J. Dunleavy, Denver; L. W. Young, Mr. Harold Sorby, Chicago; Drs. J. E. Brown, Osloosa; B. Fisher, Preston; W. R. Fullerton, Dubuque; H. M. Gillian, Mason City; J. W. Griffith, Cedar Rapids; R. R. Hammond, LeMars; S. K. Hazlek, Oelwein; C. J. Hinkley, Odebolt; S. W. Johnston, Carrol; W. G. Jones, J. A. Vincent, Shenandoah; C. B. Knowles, Glenwood; W. F. Knowles, James; P. O. Koto, Forest City; D. H. Miller, Harlan; J. J. Miller, Sioux City; S. T. Miller, Shelby; F. M. Roys, Manning; J. O. Simcoke, Stuart; H. C. Simpson, Denison; C. E. Stewart, Chariton; H. S. Stewart, Oakley; A. C. Wood, Council Bluffs, Iowa. J. H. Cock, Ottawa; G. R. Conrad, Sabetha, Kansas; S. H. Ward, St. Cloud, Minn. H. M. Burgess, South St. Joseph; L. D. Brown, Hamilton; W. A. Heck, James Wilson, St. Joseph; J. C. Milnes, Kansas City; A. B. Wilmoth, Chillicothe, Mo. B. C. Barber, W. A. Thomas, George P. Tucker, Lincoln; A. M. Blackwell, L. Clarke, S. E. Cosford, A. M. Waring, Omaha; A. Bostron, Minden; P. T. Bowers, Hastings; N. V. Byers, Osceola; J. Hagerty, Tapeer; D. Langford, Tekamah; C. A. McKim, Norfolk, Neb. Mr. W. P. Allen, Philadelphia; Drs. J. C. Foelker, Allentown; W. P. Phipps, Mr. T. J. Phipps, Lionville; Dr. B. M. Underhill, Media, Pa. Dr. R. S. Heer, Plattsville, Wis.

*Elected to Membership:* Drs. John S. Anderson (Chicago V. C.), Seward, Neb.; W. N. D. Bird (K. C. V. C.), Arkansas City, Kan.; Levi P. Beechy (Ont. V. C.), Omaha, Neb.; A. E. Behnke (Chicago V. C.), Milwaukee, Wis.; S. E. Bennett (Vet. Dept. O. S. Univ.), Kansas City, Mo.; John S. Buckley (A. V. C.), Armour Packing Co., Kansas City, Mo.; Jas. Bullivant (Ont. V. C.), Spokane, Washington; A. O. Cawley (A. V. C.), Milton, Pa.; H. A. Christmann (Vet. Dept. Univ. of Pa.), 193 Eleventh Street, Milwaukee, Wis.; P. D. Coffey (Chicago V. C.), Wellman, Iowa; Chas. E. Cotton (Vet. Dept. Univ. of Pa.), Minneapolis, Minn.; J. J. Draski (Ont. V. C.), Crete, Neb.; Fred. Evans (Ont. V. C.), Grand Island, Neb.; A. T. Everett (Ont. V. C.), Twenty-first and H Street, S. Omaha, Neb.; Edward Fox (A. V. C.), 2823 Hamilton Avenue, Baltimore, Md.; J. I. Gibson (Ont. V. C.), Denison, Iowa; Chas. Cresswell (London), 75 Montclair Street, Denver, Col.; J. W. Griffiths (Ont. V. C.), Cedar Rapids, Iowa; F. F. Hoffman (Ont. V. C.), Brookville, Pa.; J. Otis Jacobs (Univ.

of Cal. Vet. Dept.), corner of Twenty-sixth Street, N. W., Omaha, Neb.; Harry J. Kannal (Chicago V. C.), Rensselaer, Ind.; R. D. Martin (Vet. Dept. Harvard Univ.), Bridgeport, Conn.; Chester Miller (Ont. V. C.), 1124 Park Place, St. Louis, Mo.; John R. Mohler (Vet. Dept. Univ. of Pa.), Blankington Pack. Co., Milwaukee, Wis.; H. G. Moore (Vet. Dept. Ames Agr. Col.), 229 Eleventh Street, Milwaukee, Wis.: F. C. McCurdy (Vet. Dept. Univ. of Pa.), 1315 Broadway, Kansas City, Mo.; E. M. Nighbert (Ont. V. C.), Mt. Sterling, Ill.; Alex. Plummer (Chicago V. C.), Ft. Walla Walla, Washington; M. M. Poucher (Ont. V. C.), Oswego, N. Y.; John J. Repp (Vet. Dept. Univ. of Pa.), 3608 Pine Street, Philadelphia, Pa.; V. Schaefer (Chicago V. C.), Tekamah, Neb.; W. A. Shoults (Ont. V. C.), Gladstone, Manitoba; Thos. E. Smith (N. Y. C. V. S.), 309 Barron St., Jersey City, N. J.; Ray J. Stanclift (N. Y. S. V. C.), Americus, Ga.; John D. Sprague (Chicago V. C.), David City, Neb.; Wm. M. Taylor (Ont. V. C.), York, Neb.; Wm. Thompson (Chicago V. C.), Sioux City, Iowa; W. J. Tomlinson (A. V. C.), Williamsport, Pa.; S. A. Ward (Ont. V. C.), St. Cloud, Minn.; G. R. Young (Chicago V. C.), 1815 Chicago Street, Omaha, Neb.

*Reinstated:* George W. Dunphy, Quincy, Mich.; G. Ed. Leech, Milwaukee, Wis.

*Resigned:* Drs. L. McLean, 14 Nevins Street., Brooklyn, N.Y.; W. P. Stranghan, Jewett Centre, N. Y. Resignations not accepted.

Drs. S. K. Johnson, New York City, N. Y.; J. H. Wattles, Kansas City, Mo., expelled for violation of the code of ethics.

Charges against Dr. G. E. Griffin dismissed.

Editor Bell, of the *American Veterinary Review*, responded most excellently on behalf of the profession to the Mayor's genial and cordial welcome.

State Veterinarian Knowles was an interested participant in the convention's work, but could not break his slumbers early enough in the morning to hear the President's address.

Mr. Harold Sorby, of the Pasteur Vaccine Company, of Chicago, was happy in his close touch with the veterinary profession.

Even the Mayor's leather-seated chair was at the command of the members of the convention.

Manitoba was represented by Dr. W. J. Hinman, of Winnipeg, and rejoices much in the Association's new name.

Many were the expressions of sympathy for Secretary Rhoads, of the Pennsylvania State Veterinary Medical Association, who on reaching Omaha was met with the news of the death of his only child, and whose return was under such trying ordeal.

Drs. S. E. Cosford, of S. Omaha, Neb.; G. A. Scott, of Independence, Iowa, and James Vincent, Shenandoah, Iowa, were operators on cryptorchids.

Chairman Peters made several announcements as to the local reception of the ladies. Dr. M. Stalker kindly chaperoned the ladies to the Exposition on Tuesday.

State Veterinarian Clement, of Maryland, has little patience with any one who has a fixed hour for retiring or an early one for rising.

The New York State Veterinary College was well represented by the presence of Professors James Law and W. L. Williams.

Dr. T. B. Cotton, of Mount Vernon, Ohio, has become a regular attendant at convention sessions, and views with much pride the profession's advancement.

Dr. S. B. Nelson, of Pullman, Washington, was the only repre. sentative of the Pacific coast, and will wear worthily the new honor of Western Vice-President conferred so unanimously upon him.

Dr. J. C. Norton, Territorial Veterinarian of Arizona, was much interested in the subject of Texas fever. The veterinary sanitary police regulations of Arizona afford ample power for dealing with all infectious and contagious diseases.

Drs. Reynolds, Cotton, Lyford, and Ward were among the prominent veterinarians of Minnesota.

Chairman Peters, of the Committee on Diseases, reported upon actinomycosis, anthrax, black-leg, cerebro-spinal meningitis, glanders, hog-cholera, influenza, parasitic diseases, sheep-scab, osteoporosis, rabies, tetanus, Texas fever, tuberculosis, bronchitis, and strangles.

Colorado sent two of her representative veterinarians, Drs. Jos. M. Dunleavy, of Denver, and A. J. Savage, of Colorado Springs.

State Secretary Ridge, of Pennsylvania, deserves much credit for his active work, sending four applications from resident veterinarians of the Keystone State and two former practising veterinarians of Philadelphia, now engaged in Federal work in Wisconsin, more than one-seventh of the total applicants.

New Jersey's amiable representative, Dr. Lowe, of Paterson, will again "sit on the chest" for another year, receiving a unanimous re-election.

Dr. J. R. Mitchell, of Evansville, Ind., was the only representative of the Hoosier State. Evansville veterinarians have a very successful way of dealing with bad paying clients.

Some nineteen of the members-elect at Omaha were from the Central Western States, including Nebraska, Iowa, Kansas, and Missouri.

Dr. Wm. Dougherty, one of Maryland's faithful representatives of the Association, whether present or absent, never forgets his Association duties. One of the new members enters with his recommendation.

Drs. S. B. Nelson, of Pullman, Washington, and J. C. Norton, of Phœnix, Arizona, set their fellow-members a good example as distant attendants upon Association meetings, travelling several thousand miles to our annual gatherings.

Dr. C. A. Cary, of Auburn, Alabama, reported rabies in the South at Knoxville and Nashville, Tennessee, Atlanta, Ga., and Auburn, Alabama. Osteoporosis quite prevalent; pasturage the best cure. Many stables seem to have become infected centres. Dr. Cary reported upon the use of barium chloride in several cases intravenously; when temperature is above normal bad results followed.

The unanimous re-election of Secretary Stewart by a rising vote was a special tribute of appreciation of the untiring service he has given to official duty.

Editor Bell, of the *Review*, was so full of joy over the elegant banquet and the splendid responses to the various toasts, undertook to write home to his wife at an early hour of the morning, lest she should not appreciate the glorious time he was having, and it was noted that he was continually using the wrong side of the blotter, which necessitated a rewriting of his missive.

Great were the fears expressed that we should have to leave in the Midway two of our unusually staid and sober-minded members; but it was a "lambs' frolic," and the camels and feminine attractions in the dusky scenes were entrancing, and only the call of lights out and closed gates sent them back to their hotel beds, and the noonday portion of the following morning's session with dreamy eyes welcomed their return.

There were a number of faces missed from the Omaha gathering, and frequent were the inquiries after members Liautard, James and Thomas B. Rayner, Faust, Jas. L. Robertson, and others.

The deaths of Dr. D. P. Frame, of Colorado Springs, Colorado, and Dr. W. A. Giffen, of New York City, were reported, and the President directed the Committtee on Resolutions to prepare suitable record of the same.

Those who feared the discomfort of warm weather at Omaha will be surprised to learn that the light clothing carried by those from the East was not needed. On the contrary, it was to be noted that the furnishing goods and clothing stores of Omaha were frequented by the delegates in search of heavier underwear and overcoats, for all of which every one was thankful who had been at Nashville one year before.

Editor Hoskins and wife continued their Western trip to Denver, Colorado, visiting many points of interest in the snow-clad Rocky Mountains.

Delegate Dr. J. C. Foelker, of Allentown, Pa., stopped at Keokuk, Iowa, on his return trip, to visit his son, who is in practice in that city.

Veterinarian Williams again continues at the head of the Publication Committee, where he has served Association interests so well.

President Clement makes a record unknown in Association annals in the announcement of all permanent committee appointments on the acceptance of the chair.

The facilities for receiving, keeping, and displaying the complete exhibition of pathological specimens of members connected with the meat-inspection service by the Cudahy Packing Company at Omaha were very much appreciated by the convention, and well deserve the thanks of every member present.

No less than twenty-five States and Territories, the District of Columbia and Manitoba were represented at the convention. Surely a grand showing where less than ten years ago four-fifths of the membership were east of the Allegheny Mountains.

The visiting ladies desire to express through the JOURNAL columns their most sincere appreciation of the courteous attention accorded them by the ladies' reception committee.

Dr. Ayer's specimens were an object-lesson of impressive importance to even those who have not given much thought to the importance of this work.

It is said that the failure to assign Williams, of New York, and Hoskins, of Philadelphia, on the Executive Committee for the ensuing year was a peace project in the interest of maintaining the harmony that prevailed at Omaha.

Nebraska may well be proud of her attendance record at Omaha; no less than twenty-five of her resident veterinarians were present at the several sessions of the convention. Hardly a prominent member of the profession in the State was absent from the meeting.

Forty new members were added at Omaha, representing thirteen States and Manitoba; two members were reinstated.

Editor Heath, of the *Nebraska Farmer*, was an interested visitor at the convention, and was very much delighted at the proposed measures for the control of hog-cholera.

Dr. Don C. Ayer merits the thanks of every member of the profession present at Omaha for the preparation of one of the most complete exhibitions of pathological specimens gathered in the Federal meat-inspection service ever presented for examination by the profession.

Ex-President Salmon made a trip through portions of Iowa after the adjournment of the convention.

Mrs. A. T. Peters, the Misses Peters, and Mrs. Don C. Ayer, were assiduous in their attention to the lady visitors, and added a deal of pleasure to their stay in Omaha.

The attractions of the "Streets of all Nations" on the Exposition Midway and the wiles of the tropical beauties were too much for many of the delegates, and Minnesota and Pennsylvania joined hands and hearts in vieing with one another as to which could show these fair ladies the greatest attention.

The evening reception of the first day's session by the ladies of Iowa and Nebraska was an unusually enjoyable feature of the social side of our convention, and brought in closer contact all those who were present, and added another strong link to the ties that bind us together in a common work. The light refreshments around the small tables and the pleasant groups of new-made friends, the dance, and the attractive sweet music were all contributory to the closer knitting together of those engaged in our work, and the ladies who warmly and cordially second all our efforts. This feature should be maintained in all our future reunions.

Dr. C. Barnwell Robinson, of Washington, D. C., represented the United States College of Veterinary Surgeons, and thinks that Washington should be the next place of meeting.

### AT THE BANQUET.

Among others present were to be noted Drs. C. A. Cary, Auburn, Ala.; J. C. Norton, Phœnix, Arizona; C. B. Robinson, D. E. Salmon, Washington, D. C.; A. H. Baker, L. A. Merillat, Mr. Harold Sorby, of Chicago; Drs. L. W. Young, Chicago, Ill.; E. M. Nighbert, Mt. Sterling, Ill.; J. R. Mitchell, Evansville, Ind.; M. Stalker, Ames; W. G. Jones, J. A. Vincent, Shenandoah, Iowa; S. Stewart, Kansas City, Kas.; J. W. Jameson, Paris, Ky.; C. W. Heitzman, New Orleans, La.; A. W. Clement, Baltimore, Md.; Chas. E. Cotton, Minneapolis; M. H. Reynolds, St. Anthony's Park, Minnesota. J. W. Connoway, Columbia; J. C. Milnes, Kansas City, Mo. M. E. Knowles, Helena, Montana; Don C. Ayer, A. M. Blackwell, L. Clarke, S. E. Cosford, Omaha; C. M. Day, A. T. Peters, George P. Tucker, Lincoln; J. J. Drasky, Crete; V. Schaefer, Tekamah; W. M. Taylor, York, Neb. Wm. Herbert Lowe, Paterson; R. R. Bell, New York City; Wm. Henry Kelly, Albany, N. Y.; T. Bent Cotton, Mt. Vernon, Ohio; J. C. Foelker, Allentown; W. Horace Hoskins, Leonard Pearson, Philadelphia, Pa. S. B. Nelson, Pullman, Washington; H. A. Christmann, Milwaukee, Wis.

Nearly sixty covers were laid for those who participated in this very pleasant social feature of the thirty-fifth annual convention.

President Salmon sat at the head of the table, which was arranged in T shape, while the honors at the other end were presided over by the incoming President, Dr. A. W. Clement.

Ex-Secretary Morton, of the Department of Agriculture, sat at the President's left, and in responding to an impromptu toast, briefly reviewed the work of the veterinarian in connection with the Bureau of Animal Industry and the better results obtained when the veterinarians employed were placed within the classified service.

Dr. Spaulding, Health Commissioner of the city of Omaha, referred to the growing importance of the work of the veterinarian in connection with health measures of municipalities, and that

Omaha had already gained much by their employment in the better control of healthful food products for their people.

President Salmon, in a few well-chosen remarks, called attention to certain special features of the work of the Bureau, and strongly contrasted it from a health point of view as the highest attainment in sanitary science destined to preserve the health of our people, while in a commercial sense the benefits derived by those engaged in livestock industries could not be calculated.

Much difference of opinion seems to prevail as to how our annual banquet should be conducted and what should be the cost per cover.

The chef of the Millard Hotel deserves much praise for the very well-prepared spread before the banquetters. The menu was well selected; its preparation all one could desire, though he be an epicure, and the serving of the same with due appreciation of time and the gastronomic powers of the guests.

The following is a list of the specimens exhibited under the consideration of the meat-inspection question:

*Cattle.* Actinomycosis: Two heads, two tongues, lung, two livers, and glands. Tuberculosis: Lungs, pleura, liver, and lymph-glands. Texas fever: Spleen, liver, skin showing ticks, urine. Disease of the liver: Fluke, two carcass calves.

*Swine.* Cholera: Two carcasses, bowel, kidneys, lungs, and spleen. Tuberculosis: Lung, pleura, bone, lymph-glands, tongue, and heart. Abscess: By kidney worms, one carcass. Disease of kidneys: Liver hob-nail, echinococcus, inflammation. Pneumonia: Lung, liver, and heart. Skin: Tinæa (ringworm diamond.) One extra-uterine pregnancy, pig's head, one bladder, one hog stomach (filled with nails). Measles: Liver, lung, and heart, tenderloin. Cirrhosis: Liver of distillery-fed hog (one of 400, all more or less affected).

*Sheep.* Liver, lungs, and heart, caseous diseases.

The Eastern delegation of doctors en route to Omaha to attend the annual meeting of the United States Veterinary Medical Association had a rich experience while coming, and the outcome was lots of fun for them at an extra charge of $2.50 per head. Dr. W. Horace Hoskins, of Pennsylvania, was chosen chaperone of the party, and officiated in an admirable manner until Columbus, Ohio, was reached early Sunday morning, when all got off for breakfast. In returning to the car Dr. Hoskins made a mistake, and, instead of getting on the Chicago train, got on the St. Louis train, and in a twinkle both trains were moving in almost opposite directions. The doctor wore a light coat and a silk cap, his hat and all his baggage being on the Chicago train. Mrs. Hoskins was with the doctor, and her bonnet was left in the car, together with all her wearing apparel. Just what the mistake cost Dr. Hoskins in dollars and cents no one knows, and he will probably not tell, but it is probable that he had to pay well for his unfortunate blunder. The Chicago train arrived in Omaha early yesterday morning, and Dr. Hoskins' train twelve hours later. Dr. Hoskins lives at Philadelphia, and is editor of the JOURNAL OF COMPARATIVE MEDICINE AND VETERINARY ARCHIVES, and a prominent character at all conventions. The Eastern delegation will have lots of fun with him upon his arrival. He had all the sleeping-car tickets in his possession, and they had to pay for berths from Chicago to Omaha. —*The World-Herald*, Omaha, September 6, 1898.

